# Effects of Oils Rich in Linoleic and α-Linolenic Acids on Fatty Acid Profile and Gene Expression in Goat Meat

**DOI:** 10.3390/nu6093913

**Published:** 2014-09-24

**Authors:** Mahdi Ebrahimi, Mohamed Ali Rajion, Yong Meng Goh

**Affiliations:** 1 Department of Veterinary Preclinical Sciences, Faculty of Veterinary Medicine, Universiti Putra Malaysia, 43400 UPM Serdang, Malaysia; E-Mails: mehdiebrahimii@gmail.com (M.E.); gohyngmeng@gmail.com (Y.M.G.); 2 Institute of Tropical Agriculture, Universiti Putra Malaysia, 43400 UPM Serdang, Malaysia

**Keywords:** flaxseed oil, omega-3 fatty acid, gene expression, lipogenic genes, goat meat

## Abstract

Alteration of the lipid content and fatty acid (FA) composition of foods can result in a healthier product. The aim of this study was to determine the effect of flaxseed oil or sunflower oil in the goat diet on fatty acid composition of muscle and expression of lipogenic genes in the *semitendinosus* (ST) muscle. Twenty-one entire male Boer kid goats were fed diets containing different levels of linoleic acid (LA) and α-linolenic acid (LNA) for 100 days. Inclusion of flaxseed oil increased (*p* < 0.05) the α-linolenic acid (C18:3*n*-3) concentration in the ST muscle. The diet high in α-linolenic acid (*p* < 0.05) decreased the arachidonic acid (C20:4*n*-6) and conjugated linolenic acid (CLA) c-9 t-11 content in the ST muscle. There was a significant (*p* < 0.05) upregulation of PPARα and PPARγ gene expression and downregulation of stearoyl-CoA desaturase (SCD) gene in the ST muscle for the high α-linolenic acid group compared with the low α-linolenic acid group. The results of the present study show that flaxseed oil as a source of α-linolenic acid can be incorporated into the diets of goats to enrich goat meat with *n*-3 fatty acids, upregulate the PPARα and PPARγ, and downregulate the SCD gene expression.

## 1. Introduction

Dietary lipids play a significant role in the daily caloric intake of the human population in Malaysia (>26% of total calories) [1]. Modification of the fatty acid (FA) composition of foods can be a practical way to enhance the consumer’s health. However, typical Malaysian foods that are consumed have an average *n*-6:*n*-3 fatty acid ratio (FAR) of 10:1 [1].

Saturated fatty acids in meat as a source of fat in the human diet are associated with several diseases including coronary heart diseases [2]. Ruminant meat can be a good dietary source of some nutrients with health benefits, such as long chain polyunsaturated fatty acids (LC-PUFA) and conjugated linoleic acid (CLA) isomers [3,4,5]. The recommended dietary ratio of *n*-6/*n*-3 fatty acids for health benefits is 1:1–2:1 [6,7], yet the typical Malaysian diet often contains 10 or more times the amount of *n*-6 relative to *n*-3 PUFA [1]. A decrease in the levels of omega-6 or an increase in omega-3 fatty acids in the animal diets will result in the animal tissues having more favorable *n*-6 to *n*-3 ratios [8], which can lead to improved consumer health [9]. The health benefits of eicosapentaenoic (C20:5*n*-3, EPA) and docosahexaenoic (C22:6*n*-3, DHA) acids are well documented and include anti-atherogenic, antithrombotic and anti-inflammatory actions [10]. The contribution of foods of marine origin, which are rich sources of EPA and DHA, to the human diet in most parts of the world is low [11]. Therefore, alternative sources of *n*-3 PUFA are required. Development of chevon (goat meat) with enhanced levels of total *n*-3 fatty acids, through dietary supplementation with high *n*-3 oils or breeding techniques [12] would result in a considerable elevation in long chain omega-3 human intake, and provide an option to add value to goat meat.

Flaxseed oil, which contains approximately 50%–70% of α-linolenic acid (LNA) is a very rich plant source of *n*-3 fatty acids [13]. The increased levels of *n*-3 fatty acids through flaxseed feeding have been demonstrated in pork, poultry and dairy products and have helped to maintain red blood cell and plasma *n*-3 fatty acid levels in humans [14]. Biohydrogenation in ruminants leads to extensive loss of unsaturated fatty acids and the accumulation of partial hydrogenation products such as vaccenic acid (VA, C18:1 *trans* (*t*)-11) and rumenic acid (RA, C18:2 *cis* (*c*)-9,*t-*11), which have many purported health benefits [15,16]. Thus, chevon with enhanced levels of partial biohydrogenation intermediates of LNA can be produced through the feeding of flaxseed oil.

One of the best endogenous or natural activators of peroxisome proliferator-activated receptors (PPARs) are polyunsaturated fatty acids [17,18]. Many studies on mice have shown that PPARα is activated by omega-3 fatty acids [19] but their lipid metabolism is different from that in ruminants. Several genes that encode for several enzymes, which are involved in carbohydrate and lipid metabolism are suppressed by both *n*-3 and *n*-6 PUFA but not by saturated, trans and monounsaturated fatty acids (MUFA) [20,21]. In ruminants, the transcription factors Sterol Regulatory Element-Binding Proteins (SREBP1) [22,23], PPAR-α [23] and PPAR-γ [22,23] regulate the expression of the stearoyl-CoA desaturase (SCD) gene. The omega-6 and omega-3 fatty acids are also important as they play a vital role in the cellular activities, metabolism and nuclear events that manage gene transcription [24].

The present study was conducted to determine the effect of flaxseed oil or sunflower oil in the goat diet on the fatty acid composition and PPARα, PPARγ and Stearoyl CoA desaturase (SCD) gene expression in the ST muscle.

## 2. Experimental Section

### 2.1. Animal Welfare

This study was undertaken following the guidelines of the Research Policy on Animal Ethics of the Universiti Putra Malaysia.

#### 2.1.1. Animals, Diets, and Management

Twenty-one 5-month-old entire male Boer kid goats with an initial body weight (BW) of 13.66 ± 1.07 kg were allocated in equal numbers to three different dietary treatments using a completely randomized design and supplemented with different inclusion levels of *n*-6:*n*-3 FAR. Goats were housed individually in wooden pens measuring 1.2 m × 1 m each, built inside a shed with slatted flooring 0.5 m above the ground. The experimental diets were equivalent in metabolizable energy and protein being 2.51 Mcal/kg and 13% crude protein, respectively [25]. The diets were based on 70% concentrate and 30% oil palm frond silage (Table 1). The rations were mixed and fed *ad libitum*. Drinking water and mineral blocks were provided *ad libitum*. The goats were allowed a 3 week adjustment period and were then fed for 100 days.

The experimental diets prepared contained either low *n*-3, using sunflower oil without the flaxseed oil as the control group (LLNA), medium *n*-3, using sunflower oil + flaxseed oil (MLNA) or high *n*-3 (HLNA) using flaxseed oil. The flaxseed oil was used as the main source of α-linolenic acid (C18:3*n*-3) while sunflower oil was used as the main source of linoleic acid (C18:2*n*-6) (Table 1). The animals were fed twice daily at 3.7% of BW (DM basis). The diets were adjusted to be isonitrogenous and isocaloric and to meet the energy and protein requirements of growing goats. At the end of the feeding period all of the goats were slaughtered after an overnight fast (12 h). Samples of the distal region of *semitendinosus* muscle were taken and frozen at −80 °C for fatty acid analysis. The ST muscle was quickly excised immediately after slaughter and snap-frozen in liquid nitrogen and stored at −80 °C until RNA extraction.

#### 2.1.2. Chemical Analyses

Feed samples (500 g) were collected every 7 day and stored at 4 °C. Refusals of each goat were weighed daily and stored at 4 °C until analyzed for dry matter (DM). Feed samples were dried at 60 °C for 48 h to determine the DM content, ground to pass a 1-mm screen and analyzed for crude protein (CP), ether extract (EE), ash and organic matter (OM) according to standard methods [26]. Crude protein (CP, total nitrogen × 6.25) was determined by the method (number 990.03) of the (AOAC) [26]. Neutral detergent fiber (NDF) and acid detergent fiber (ADF) were determined according to [27].

**Table 1:** Ingredients and chemical composition of the experimental diets.

Diets	LLNA	MLNA	HLNA
**Ingredients (% of DM)**			
Oil palm frond silage	30.00	30.00	30.00
Corn, grain	17.00	17.00	17.00
Soybean meal	13.30	13.30	13.30
Palm kernel cake	25.11	25.11	25.11
Rice bran	8.18	8.18	8.18
Flaxseed oil	0.00	0.40	1.30
Palm kernel oil	1.10	1.00	0.10
Sunflower oil	2.30	2.00	2.00
Mineral premix	0.50	0.50	0.50
Vitamin premix	0.50	0.50	0.50
Ammonium chloride	1.00	1.00	1.00
Limestone	1.00	1.00	1.00
**Chemical Composition**			
ME (Mcal/Kg) ^1^	2.51	2.51	2.51
CP%	13.00	13.00	13.00
EE%	7.00	7.00	7.00
NDF%	48.90	48.90	48.90
ADF%	33.00	33.00	33.00
Ca%	0.68	0.68	0.68
P%	0.36	0.36	0.36
**Fatty Acid Composition (% of Total Identified Fatty Acids)**			
C10:0, Capric	0.90	0.90	0.53
C12:0, Lauric	7.04	7.12	3.53
C14:0, Myristic	3.04	3.73	1.79
C16:0, Palmitic	16.14	15.36	15.65
C16:1, Palmitoleic	0.22	0.24	0.21
C17:0, Margaric	0.29	0.28	0.29
C18:0, Stearic	5.85	5.88	5.68
C18:1 *n*-9, Oleic	27.40	27.62	27.78
C18:2 *n*-6, Linoleic	35.68	32.40	30.92
C18:3 *n*-3, α-Linolenic	3.44	6.47	13.63

The result is the mean of five replicates for each treatment. LLNA: low *n*-3 FA; MLNA: medium *n*-3 FA; HLNA: high *n*-3 FA; ^1^: Calculated values.

#### 2.1.3. Measurement of FA

Total FA were extracted from the oils, experimental feeds, and ST muscle using the method of [28], modified by [29] as described by [30]. Fatty acid methyl esters (FAME) of the extracted fatty acids were prepared using the methods by AOAC (1990) [26]. The FAME was separated by gas liquid chromatography on an Agilent 7890A GC system (Agilent, Palo Alto, CA, USA) using a 100 m × 0.25 mm ID (0.20 μm film thickness) Supelco SP-2560 capillary column (Supelco, Inc., Bellefonte, PA, USA) as described by [30]. The FA concentrations are expressed as % total identified FA. A reference standard (mix C4-C24 methyl esters; Sigma-Aldrich, Inc., St. Louis, MI, USA), CLA standard mixture (O-5507 Sigma-Aldrich, Inc., St. Louis, MI, USA) and CLNA standard mixture (47792 Supelco, Chemical Co., St. Louis, MO, USA) was used to determine recoveries and correction factors for the determination of individual FA. The CLA and CLNA isomers which were not included in the standard mixtures were identified by specific standards as described in the literature [31,32].

#### 2.1.4. Tissue Collection and RNA Extraction and Purification and Real-Time Polymerase Chain Reaction (PCR)

Total RNA was extracted from 100 mg of frozen tissue using the RNeasy^®^lipid tissue mini kit (Cat. No. 74804, Qiagen, Hilden, Germany) and DNase digestion was completed during RNA purification using the RNase-Free DNase set (Qiagen, Hilden, Germany) according to the manufacturer’s instructions. Total RNA concentration and purity was evaluated by NanoDrop ND-1000 UV-Vis Spectrophotometer (NanoDrop Technologies, Wilmington, DE, USA) using the 260/280 nm ratio of absorbance. Purified total RNA (1 μg) was reverse transcribed using a Quantitect^®^ reverse transcription kit (Qiagen, Hilden, Germany). Real-time PCR was performed with the Bio-Rad CFX96 Touch (Bio-Rad Laboratories, Hercules, CA, USA) using optical grade plates using Quantifast^®^ SYBR green PCR kit (Cat. no. 204054, Qiagen, Hilden, Germany). The sequences of primers are shown in Table 2.

**Table 2:** Primers used in this study.

Target Group		Sequence 5′—3′	Length, nt	Reference
**β-actin**	F	CGC CAT GGA TGA TGA TAT TGC3	123	[23]
	R	AAG CGG CCT TGC ACA T3		
**PPARα**	F	TGC CAA GAT CTG AAA AAG CA	101	[33]
	R	CCT CTT GGC CAG AGA CTT GA		
**PPARγ**	F	CTT GCT GTG GGG ATG TCT C	121	[33]
	R	GGT CAG CAG ACT CTG GGT TC		
**SCD**	F	CCC AGC TGT CAG AGA AAA GG		
	R	GAT GAA GCA CAA CAG CAG GA	115	[33]

F: forward; R: reverse.

The reference gene employed to normalize the tested genes was β-actin. The primers were purchased through 1st BASE oligonucleotide synthesis (1st Base, Singapore). The integrity and concentration of RNA and real time-PCR were assessed as described previously by Ebrahimi *et al*., (2013) [34].

### 2.2. Data Analysis

Results were analyzed using analysis of variance with the different treatments as the main effects. FA data and all the gene expression data were analyzed by one-way ANOVA, using the MIXED procedure of the SAS software package, Version 9.1 (SAS Inst. Inc., Cary, NC, USA). Means were separated using the “PDIFF” option of the “least-squares means (LSMEANS)” statement of the MIXED procedure. Differences of *p* < 0.05 were considered to be significant. The data were checked for normality using the UNIVARIATE procedure of SAS software (SAS Inst. Inc., Cary, NC, USA) and the results in the tables are presented as means and pooled standard error of the mean.

## 3. Results and Discussion

### 3.1. Nutrient and Fatty Acid Composition of Experimental Diets

The diet compositions are summarized in Table 1. The crude protein content met the goat requirements [25]. Flaxseed oil contained the highest amount of α-linolenic acid and sunflower oil contained the highest amount of linoleic acid. Linoleic acid (LA, C18:2*n*-6) was the most abundant fatty acid in the LLNA (35.68% of total fatty acids) and MLNA (32.40% of total fatty acids) diets, followed by C18:1*n*-9 and C16:0. The LNA in the HLNA diets was higher than the LLNA and MLNA diets.

### 3.2. Fatty Acid Composition of ST Muscle

#### 3.2.1. *n*-3 and *n*-6 Polyunsaturated Fatty Acids

The percentage of LNA in the ST intramuscular fat increased (*p* < 0.05) with the inclusion of flaxseed oil in the diet. The increase in LNA was also reported for cattle [35] and dairy goats [36]. Infusion of LNA post-rumenally can produce a considerably greater LNA enrichment of the milk fat [37]. In the present study, the dietary concentrations of C18:2*n*-6 was higher in the LLNA group (35.68%) compared to the HLNA group (30.92%).

The inclusion of LNA from flaxseed oil in the diet increased the levels of C20:5*n*-3 (*p* < 0.05). The increased availability of LNA would result in an increased synthesis of C20:5*n*-3 by chain elongation and desaturation [38,39]. The differences between ruminant tissues are still being investigated as there are differences in the rates of elongation and desaturation between species and tissues.

The reduced C20:4*n*-6 (*p* < 0.05) through feeding flaxseed oil was also observed in beef cattle [39]. These changes in *n*-6 PUFA in the muscle were attributed to the competition between LA and LNA for the same elongation and desaturation enzymes.

The LNA concentration was highest in the ST muscle (2.00%) of goats fed the flaxseed oil diet. The concentrations of C20:5*n*-3, C22:5*n*-3 and C22:6*n*-3, which are metabolic products of LNA, also followed a similar response (*p* < 0.05) to LNA concentrations. Jerónimo *et al*., (2009) [40] and Mapiye *et al*., (2013) [35] had reported that the concentration of C20:5*n*-3, C22:5*n*-3 and C22:6*n*-3 increased in cattle muscle when fed with high levels of flaxseed oil rather than sunflower oil. The limited capacity of the conversion of C18:3*n*-3 of health promoting *n*-3 LC-PUFA humans [41] stresses the importance of its supply through the diet. This could be due to the probable competition between C18:2*n*-6 and C18:3*n*-3 for desaturation and elongation enzymes, which affect the conversion to their long chain fatty acid derivatives [42]. The addition of fish oil to ruminant diets has been shown to be more effective to elevate the EPA and DHA in the meat rather than lipid sources rich in C18:3*n*-3 like flaxseed oil [43,44]. However, using fish oil may decrease the meat shelf life and affect the flavor [45], as well as on the sustainability of increased use of fish oil in the food chain [8]. The LLNA treatment showed less *n*-3 LC-PUFA rather than the HLNA treatment. The concentration of C18:2*n*-6 in muscle lipids was higher for LLNA goats, suggesting that inhibition of the C18:2*n*-6 metabolism might occur. This inhibition may occur by repression of the gene expression mediated by C18:2*n*-6 [46].

Decreasing the dietary *n*-6:*n*-3 FAR in the goat diet by 4.58 fold (from 10.38 to 2.27; Table 1) with supplementation of oil altered that same ratio by 3.80 fold in the ST meat from 11.67 to 3.07. When high concentrate diets are fed, replacing supplement oil having a high amount of C18:2*n*-6 with one having a high amount of C18:3*n*-3, such as flaxseed oil, the *n*-6:*n*-3 FAR can be decreased to half in meat, thus making it a healthier meat for hypercholesterolemic or diabetic people. The *n*-6:*n*-3 FAR in the food which should range between 1 and 2 [7] was 3.07 in the HLNA treatment, In the LLNA treatment, 50.25% of *n*-3 PUFA were *n*-3 LC-PUFA and this is important as the health benefits of *n*-3 FA are mostly associated with the *n*-3 LC-PUFA and also the metabolism of C18:3*n*-3 in humans is limited [41].

#### 3.2.2. Triene and Diene Biohydrogenation Products

Dietary PUFA can be biohydrogenated by rumen microorganisms [47]. Feeding flaxseed oil increased the total CLNA (*p* < 0.05, Table 3). The CLNA isomer is formed as an intermediate during LNA biohydrogenation. Of the individual CLA isomers, rumenic acid (RA; CLA c-9 t-11) was the major isomer and the LLNA diet produced the highest levels of RA where the high LA diet was fed to goats. Similarly, significantly higher levels of RA were also found in beef from cattle fed sunflower seed compared to flaxseed [35].

Both C18:1 t-11 and CLA c-9 t-11 concentrations in the ST muscle tended to decrease with increasing dietary flaxseed oil with the latter decreasing dramatically in goats fed the HLNA diet. This result may be explained by the increase in C18:2*n*-6 in goats fed the 10.38:1 *n*-6:*n*-3 FAR diet, where more C18:2*n*-6 was isomerized to CLA c-9 t-11 and hydrogenated to C18:1 t-11 in the rumen of goats fed the LLNA leading to more deposition in the muscle. Our present data confirmed an earlier observation of [40] who reported increased concentrations of C18:1 t-11 and CLA c-9 t-11 in the *semitendinosus* muscle of the lamb when sunflower oil was replaced by linseed oil as the fat supplement in the diet. The synthesis of these isomers from C18:2*n*-6 may have been more efficient than that from C18:3*n*-3. At least for forage-based diets, C18:2*n*-6 ruminal biohydrogenation occurs with an initial isomerization with the formation of CLA c-9 t-11 and its reduction of C18:1 t-11. The biohydrogenation of C18:3*n*-3 results in more diverse products including C18:3 c-9 t-11 c-15, C18:2 t-11 c-15, C18:1 t-15, C18:1 c-15 and C18:1 t-11, but not CLA c-9 t-11. Therefore, sunflower oil supplementation would produce more rumen-derived CLA c-9 t-11 than flaxseed oil [47]. Noci *et al*., (2007) [48] observed a higher CLA c-9 t-11 content in the ST muscle of heifers supplemented with sunflower oil than with flaxseed oil. This can be explained by the fact that most of the CLA c-9 t-11 present in tissues is derived from endogenous desaturation of C18:1 t-11 [49], which originates during ruminal biohydrogenation of both C18:2*n*-6 and C18:3*n*-3. However, the CLA c-9 t-11 is also synthesized by direct isomerization of C18:2*n*-6 in the rumen.

**Table 3:** Fatty acid composition (percentage of total identified fatty acids) of the *semitendinosus* (ST) muscle in Boer goats fed diets with different levels of flaxseed oil.

ST	LLNA	MLNA	HLNA	SE	*p*-Value
C10:0, Capric	0.35	0.33	0.32	0.02	0.75
C12:0, Lauruic	3.55	3.31	3.15	0.22	0.53
C14:0, Myristic	3.53	2.69	1.39	0.23	0.04
C14:1, Myristoleic	0.36	0.58	0.29	0.10	0.05
C15:0, Pentadecanoic	0.44	0.59	0.59	0.08	0.55
C15:1, Pentadecenoic	0.41	0.36	0.40	0.10	0.16
C16:0, Palmitic	21.55	21.63	20.31	1.35	0.14
C16:1 *n*-7, Plamitoleic	1.64	1.94	1.53	0.14	0.46
C17:0, Margaric	1.00	1.16	0.92	0.23	0.29
C17:1, Margaroleic	1.09	0.75	0.70	0.10	0.51
C18:0, Stearic	12.00	12.44	11.54	0.68	0.06
C18:1 *n*-9, Oleic	34.66	36.16	40.77	1.76	0.03
C18:1 t*-*11 Vaccenic	1.99	1.33	0.95	0.17	0.04
C18:2*n*-6, Linoleic	10.92	9.99	9.47	0.33	0.02
CLA c-9 t-11	1.22	0.77	0.50	0.12	0.001
CLA c-12 t*-*10	0.53	0.46	0.42	0.06	0.07
C18:3*n*-3, α-Linolenic	0.54	0.87	2.00	0.11	0.001
CLNA c-9,t-11,c-15	0.28	0.33	0.41	0.03	0.03
CLNA c-9,t-11,t-15	0.03	0.05	0.07	0.01	0.18
C20:4 *n*-6, Arachidonic	3.51	3.31	2.73	0.17	0.02
C20:5 *n*-3, Eicosapentaenoic	0.28	0.67	0.74	0.07	0.005
C22:5*n*-3, Docosapentaenoic	0.05	0.04	0.07	0.01	0.05
C22:6*n*-3, Docosahexaenoic	0.38	0.63	1.21	0.08	0.04
SFA	42.42	42.14	38.22	1.63	0.04
MUFA	40.15	41.12	44.64	1.80	0.02
PUFA*n*-3	1.24	2.21	4.03	0.20	0.001
PUFA*n*-6	14.43	12.88	12.20	9.04	0.01
Total PUFA	15.68	15.09	16.23	0.90	0.06
Total *trans* FA	1.99	1.33	0.95	0.17	0.01
Total CLA	1.75	1.23	0.92	0.14	0.001
Total CLNA	0.31	0.38	0.48	0.03	0.04
*n*-6:*n*-3 FAR	11.67	6.25	3.07	0.42	0.03
PUFA:SFA Ratio	0.37	0.37	0.43	0.02	0.57

LLNA: low *n*-3 FA; MLNA: medium *n*-3 FA; HLNA: high *n*-3 FA; SFA = sum of C10:0 + C12:0 + C14:0 + C15:0 + C16:0 + C17:0 + C18:0; MUFA = sum of C14:1 + C16:1 + C17:1 + C18:1*n*-9. *n*-3 PUFA = sum of C18:3*n*-3 + C20:5*n*-3 + C22:5*n*-3 + C22:6*n*-3. *n*-6 PUFA = sum of 18:2*n*-6 + 20:4*n*-6; Total CLA = sum of CLA c-9 t-11 + CLA c-12 t-10; Total CLNA = sum of CLNA c-9,t-11,c-15 + CLNA c-9,t-11,t-15; *n*-6:*n*-3 FAR = (C18:2*n*-6 + C20:4*n*-6) ÷ (C18:3*n*-3 + C20:5*n*-3 + C22:5*n*-3 + C22:6*n*-3).

In the present study, the flaxseed oil supplementation has increased the total CLNA due to alterations in the concentration of CLNA c-9 t-11 c-15 while there was no significant effect for the CLNA c-9 t-11 t-15 isomer. The level of CLNA c-9 t-11 c-15 was increased by feeding the HLNA compared to the LLNA diet (*p* < 0.05) and an increase was observed when flaxseed oil was included in the diet (*p* < 0.05). Feeding flaxseed has been previously shown to increase CLNA c-9 t-11 c-15 in beef [16,35]. Combined, these results along with ours indicate that feeding flaxseed oil will result in greater accumulations of initial intermediates of LNA biohydrogenation.

#### 3.2.3. Monounsaturated Fatty Acids (MUFA)

In the ST, total MUFA were significantly higher with HLNA compared to the LLNA feeding (*p* < 0.05). Levels of C16:1n-7 were not affected by the dietary treatments (*p* > 0.05). Although Aharoni *et al*., (2004) [50] and Bartoň *et al*., (2007) [51] did not observe changes in muscle C18:1*n*-9 when flaxseed was added to beef diets, Mapiye *et al*., (2013) [35] reported an increase in C18:1*n*-9. In the present study, it would appear that LNA and/or its biohydrogenation products may have stimulated C18:1*n*-9 synthesis. Total vaccenic acid (VA; C18:1t-11) in intramuscular fat was influenced by the oil types, with the highest level found in the LLNA diet (Table 3). The increase in C18:1t-11 in both intramuscular and subcutaneous fat with sunflower seed supplementation was also higher in beef [35].

#### 3.2.4. Saturated Fatty Acids (SFA)

The reduction of total SFA by feeding flaxseed oil (*p* < 0.05) was mainly due to a reduction in C14:0. Although the levels of C14:0 changed with the oil type in the diet (*p* < 0.05) the levels of C12:0, C16:0 and C18:0 did not show significant changes (*p* > 0.05). Scollan *et al*., (2001) [39] also found feeding flaxseed to steers had no significant effects on C18:0. Bartoň *et al*., (2007) [51] and Nassu *et al*., (2011) [16] also reported negligible changes in C16:0 with no change in C18:0 in the intramuscular tissue of flaxseed-fed heifers. The decreases in all cases are due to the inhibitory effect of LNA and/or its biohydrogenation products on *de novo* fatty acid synthesis.

#### 3.2.5. Nutritional Quality of Meat Fatty Acids

According to the Food and Nutrition Board (2005) [52] an acceptable intake (AI) for omega-3 is 1.6 g/day for men and 1.1 g/day for women [52]. Additionally, the *n*-3 fatty acids in this study were calculated as mg per 100 g of serving ST meat to estimate if the ST from the goats in the present study fulfill the level necessary to make a *n*-3 fatty acid source required (the required level is 1.1 to 1.6 g/day). However, feeding flaxseed oil increased significantly (*p* < 0.05) the total omega-3 fatty acid content in the meat, but the lean meat did not obtain the required level (only 19.99–55.51 mg *n*-3 fatty acids per 100 g meat). Therefore, other sources of *n*-3 fatty acids should be provided to fulfill the acceptable intake requirement for human.

The *n*-6:*n*-3 FAR is highly influenced by the FA composition of the diet fed to the animals [53]. Consistently, the decline in the dietary *n*-6:*n*-3 FAR, due to supplementation of flaxseed oil in the diets, the *n*-6:*n*-3 FAR of intramuscular fat also declined. The HLNA resulted in *n*-6:*n*-3 FAR below 4, which is the maximum recommended level for human by Simopoulos (1994) [7].

Increasing vaccenic acid (VA) in the human diet can reduce pro-inflammatory cytokines [54,55]. It can have beneficial health effects through Δ-9 desaturation to rumenic acid (RA; CLA c-9 t-11), which may decrease the risk of cancer and heart related disease, modulate immune inflammatory responses and enhance bone mass [56]. In this study, the calculated levels of VA in ST muscle from goat fed flaxseed oil and sunflower oil containing diets would be 13.05 and 31.50 mg per 100 g ST, respectively, while the RA contents would be 6.84 and 18.41 mg per 100 g ST muscle, respectively.

### 3.3. Effect of Dietary n-6:n-3 FAR on mRNA Expression of PPARα, PPARγ and SCD in ST Muscle

**Figure 1:**
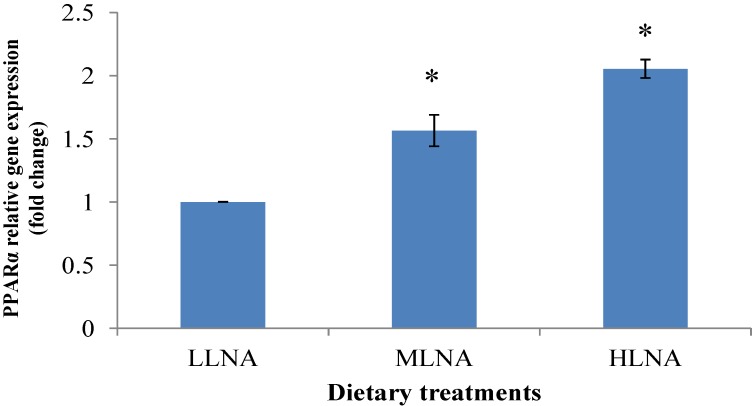
Comparisons of peroxisome proliferator-activated receptor α (PPARα) in the ST muscle of goats fed diets with different *n*-6 or *n*-3 FA. Values indicated by * show significant difference compared with LLNA group (*p* < 0.05). LLNA: low *n*-3 FA, MLNA: medium *n*-3 FA and HLNA: high *n*-3 FA. Error bar = 1 SE.

**Figure 2:**
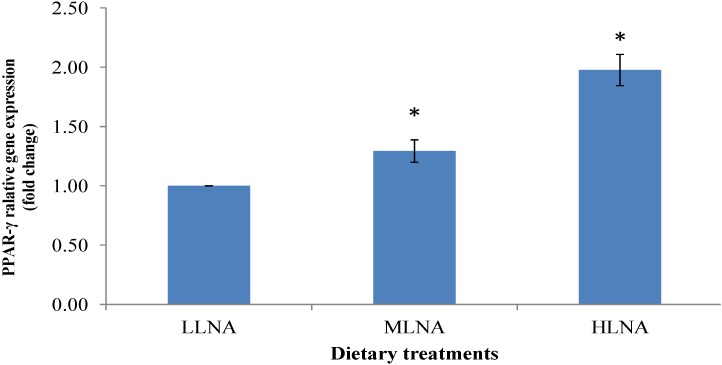
Comparisons of PPARγ in the ST muscle of goats fed diets with different *n*-6 or *n*-3 FA. Values indicated by * show significant difference compared with LLNA group (*p* < 0.05). LLNA: low *n*-3 FA, MLNA: medium *n*-3 FA and HLNA: high *n*-3 FA. Error bar = 1 SE.

**Figure 3:**
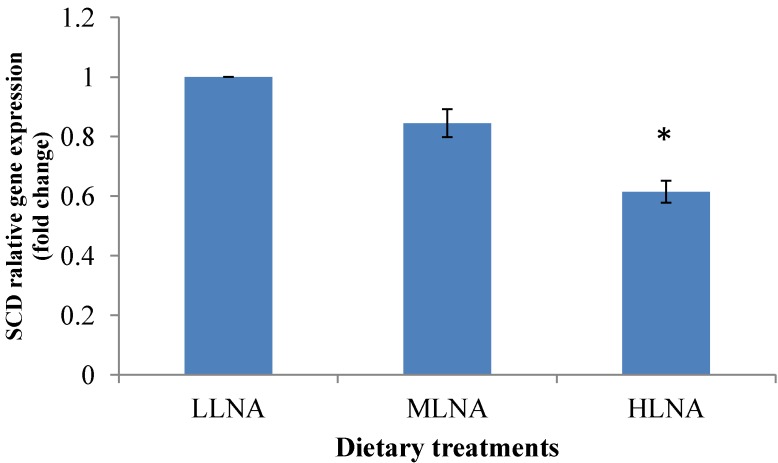
Comparisons of stearoyl-CoA desaturase (SCD) in the ST muscle of goats fed diets with different *n*-6 or *n*-3 FA. Values indicated by * show significant difference compared with the LLNA group (*p* < 0.05). LLNA: low *n*-3 FA, MLNA: medium *n*-3 FA and HLNA: high *n*-3 FA. Error bar = 1 SE.

Figure 1, Figure 2 and Figure 3 show the relative gene expression in the ST muscle of the HLNA group compared to LLNA group. The PPARα and PPARγ genes showed a higher level of expression in the HLNA group compared with the LLNA group (*p* < 0.05, Figure 1), indicating that increasing the dietary LNA had upregulated the PPARα and PPARγ gene expression.

The SCD gene expression showed a significant (*p* < 0.05, Figure 3) reduction in the HLNA group compared to the LLNA group suggesting that the SCD gene was downregulated by the flaxseed oil treatment.

The objectives of the this experiment, which was to explain whether the different oils could affect the expression of mRNA encoding for PPARs and SCD in goat tissues, were achieved by the present results. Different oils in the goat feed altered the PPARα and PPARγ expression in the goat tissues where the addition of flaxseed oil increased the PPARα and PPARγ expression in the HLNA and MLNA treatment groups compared to the LLNA group. The PPARα responds to changes in dietary fat by activating the expression of various enzymes involved in fatty acyl CoA formation and hydrolysis, fatty acid elongation and desaturation, and fatty acid oxidation [57]. The significant differences in the mRNA expression of PPARα, PPARγ and SCD in the ST muscle between different dietary treatments suggest that the principal pathways through which fatty acids act to modulate the expression of lipogenic genes is through the altered expression of PPARα, PPARγ and SCD. Al-Hasani and Joost (2005) [58] also showed that lowering the *n*-6:*n*-3 FAR in the rodent diet can increase PPARγ activity in target tissues, which are associated with increased insulin sensitivity. Therefore, based on these results, it could be speculated that feeding HLNA can increase the PPARγ expression in chevon. The results of the current study clearly support the existence of a relationship between the PPAR gene expression and intake of the *n*-3 fatty acids.

Several nutrients such as fatty acids, carbohydrates, hormones [23,59] and cholesterol [60] strongly modulate the expression of SCD. The observation that the flaxseed oil downregulated the SCD gene expression support an earlier report by Deiuliis *et al*., (2010) [61] who also reported a reduced SCD gene expression in the tissues of Angus steers fed flaxseed rich in α-linolenic acid compared with control diets without flaxseed. The downregulation in mRNA levels was probably due to the high concentrations of α-linolenic acid in the flaxseed compared with the control diet. Waters *et al*., (2009) [23] found a negative relationship between SCD gene expression and *n*-3 PUFA, EPA (C20:5n-3), DHA (C22:6*n*-3) and α-linolenic acid (C18:3*n*-3) in beef cattle, which is in agreement with our present findings where the downregulation of SCD occurred in the HLNA treatment group with the highest α-linolenic acid concentrations.

## 4. Conclusions

The results of the present study showed that goats fed diets supplemented with high α-linolenic acid had increased omega-3 fatty acids in their meat, an upregulation of the PPAR-α, PPAR-γ and downregulation of the SCD gene compared to those fed a diet supplemented with high linoleic acid. The results also showed that different dietary fats led to different levels of PPAR-α, PPAR-γ and SCD gene expression in the goat muscle. Overall, flaxseed oil in the goat diet had beneficial effects and such supplementation could improve the quality of the chevon by significantly increasing the muscle omega-3 fatty acid concentration, making it a healthier product.
